# Occupational Exposure and Economic Inequity Along a Game Meat Trade Network in Cross River State, Nigeria

**DOI:** 10.3390/ani16111666

**Published:** 2026-05-29

**Authors:** Katharine E. T. Thompson, Christian E. Herrera, Wilfred A. Ayambem, Alobi O. Alobi, Nzube M. Ifebueme, Oshama M. Otukpa, Tomilola F. Aderibigbe, Matthew R. Keenan, Sagan R. Friant

**Affiliations:** 1 Department of Anthropology, Pennsylvania State University, 228 Susan Welch Building, University Park, PA 16802, USA; ceh364@psu.edu (C.E.H.); mrk5708@psu.edu (M.R.K.); 2 Huck Institutes of the Life Sciences, Pennsylvania State University, University Park, PA 16802, USA; 3 Smeal College of Business, Pennsylvania State University, University Park, PA 16802, USA; 4 Department of Forestry and Wildlife Resources Management, University of Calabar, Calabar 540281, Nigeriamarijayokoi.mj@gmail.com (O.M.O.);

**Keywords:** West Africa, bushmeat, zoonotic disease, supply chain, food safety, market dynamics

## Abstract

Game meat supply chains create variable safety risks and economic benefits across traders, yet research often focuses on hunters or consumers while overlooking meat preparation and processing roles. This study examines how zoonotic disease exposure, precautionary behaviors, and economic returns vary across hunters, vendors, and restaurant workers within the game meat trade in Cross River State, Nigeria. Between January 2023 and April 2024, we conducted 91 annual and 760 monthly interviews with 91 actors, including hunters, vendors, and restaurant workers, documenting handling practices, hygienic precautions, risk perceptions, and income derived from the game meat trade. Hunters reported the highest levels of direct contact with high-risk meat states and the lowest use of hygienic precautions. Vendors generated higher income than other actors, while hunters reported lower economic dependence on game meat than either vendors or restaurants. These findings demonstrate that game meat safety risks and livelihood benefits are distributed unevenly across value chains. Recognizing these asymmetries is essential for designing food safety interventions and policies that protect public health and anticipate differential impacts on livelihoods.

## Simple Summary

Game meat is an important source of food and income for many people in Nigeria, but handling carcasses can also expose people to diseases from animals. Most research has focused on hunters or consumers, while giving less attention to the many people who butcher, transport, sell, and prepare meat along the way. This study examines how health risks, protective behaviors, and economic returns vary across hunters, vendors, and restaurant workers within the game meat trade in Cross River State, Nigeria. We found that hunters most often handled raw and undercooked meat while also using the fewest protective measures, while vendors earned more income than other individuals. Restaurant workers and vendors, although often viewed as further removed from slaughter, were still regularly exposed to unpreserved meat. These findings show that health risks and economic benefits are not shared equally across the trade and suggest that interventions should be tailored to different roles to better protect both public health and livelihoods.

## 1. Introduction

The butchery and distribution of game meat—like any domestic meat industry—poses food safety risks to hunters, consumers, and supply chain workers. Handling meat from animals can result in spillover events—the transmission of a pathogen from an animal to human host [1,2,3,4]. Globally, 75% of emerging infectious diseases originate from such spillover events, and a majority of these originate from wildlife species [5,6,7]. In Nigeria, notable public health threats such as tuberculosis, trypanosomiasis, toxoplasmosis, taeniasis, rabies, Lassa fever, and yellow fever have wildlife origins [8]. Several top-ranked national disease priorities such as Mpox, Ebola virus disease, and anthrax, have circulated in some of the same wildlife species that are frequently handled for consumption [9]. Outbreaks of cutaneous anthrax, listeria, and other diseases have been linked to risky meat handling and lack of hygienic precautions [10,11]. Yet surveillance procedures and risk assessment frameworks in the game meat sector remain underapplied compared to those used in other food industries [12,13]. Given this, understanding how people interact with wildlife through capture, processing, and resale is crucial to identifying points of heightened zoonotic exposure and opportunities for targeted intervention.

A value-chain approach considers the game meat industry in its totality: an interdependent network of occupational roles across which risk and benefit are not evenly distributed. In general, supply chain models encompass all the activities a business undertakes to create a product, from sourcing raw materials to the delivery of consumable goods [14,15]. The economic benefit that traders receive from their participation in the industry (e.g., income from game meat sales) varies with their stage in the supply chain (whether early or late) and the actions they take to modify the meat before passing it to the next actor in the chain. In a value-added supply chain, the product is assumed to be iteratively refined in ways that adds value at each step in the process [15]. Game meat is traded within value-added supply chains that vary in length and complexity, generally involving multiple actors who handle and process the meat between harvest to consumption [16]. In this context, value is added not only through processing and preparation, but also through movement away from the point of harvest as meat passes between different actors in the chain. Among these roles are hunters who capture and slaughter wildlife; vendors who buy and resell meat to other traders whether in roadside, market, or mobile contexts; and restaurants that sell food products directly to consumers. How individuals self-identify within these systems is highly variable, yet many studies consider benefits and risks for either hunters, vendors, or consumers in isolation (e.g., Alhaji et al., 2018 [17]; Andong et al., 2023 [18]; Froese et al., 2023 [19]; McNamara et al., 2016 [20]; Subramanian, 2012 [21]). Other studies focus on roles within one aspect of the broader rural-urban spectrum (e.g., Saylors et al., 2021 [22]). While these approaches offer an in-depth understanding of specific roles, they have a limited ability to compare risk and benefit across the entire supply chain.

Understanding trade-role-level variation in vulnerability to health risks and economic shocks (e.g., interruptions in supply, reductions in demand, etc.) is crucial for designing effective and equitable risk mitigation strategies [23]. Supply chain economics suggest that those closest to the source—e.g., miners in the diamond industry or hunters in the game meat trade—have less bargaining power and fewer resources than those further along the chain [24,25]. While each participant in the supply chain generally aims to maximize profits and minimize risks, there remains a structural imbalance. Unlike the domestic meat sector, game meat handling is less regulated and faces less guidance on precautionary practices [12]. The cryptic nature of the game meat trade—where wildlife harvests are criminalized or stigmatized—complicates our understanding of hygiene-related risks by driving meat handling underground where visibility to surveillance efforts and access to hygiene amenities may be further reduced [26]. If game meat handling expands and accelerates across Africa, workers will be increasingly at risk due to low awareness of health hazards, high contact with risky meat states (e.g., fresh carcasses during slaughter and butchery), and low access to personal protective equipment [27]. Those responsible for sourcing wildlife—particularly hunters and other early-stage actors—may shoulder the greatest health risks while receiving relatively low economic returns. In this way, individuals who are already disempowered may face compounded vulnerability, lacking the capacity to buffer against and recover from shocks to their livelihoods [28,29,30,31].

Nigeria is a well-known hub within the game meat industry, where wildlife harvesting and trade provide an important source of income and food security for thousands of households [18,32,33,34,35,36]. Yet despite the scale and importance of this sector, relatively little is known about how relative risks and benefits are distributed among those who participate in the trade. We hypothesize that upstream actors, particularly hunters, may bear the highest exposure risk while receiving the lowest economic returns, whereas downstream actors may receive greater economic benefits while facing comparatively lower exposure risk.

Therefore, the objective of this study is to evaluate how zoonotic exposure risk, precautionary behaviors, and economic returns vary across actors within the game meat value chain in Cross River State, Nigeria. To investigate this, we focused our inquiry along three interrelated dimensions: supply chain structure, occupational risk and risk perception, and financial benefit. First, we apply a value chain framework to characterize participation across the game meat trade. Second, we examine how zoonotic exposure risk varies along this chain through differences in meat handling practices, injuries, and duration of exposure. We then assess how actors perceive these risks and adopt precautionary behaviors to protect themselves. Lastly, we consider these factors alongside measures of wealth to understand how health hazards and economic rewards are balanced between actors within the trade. Together, this approach allows us to generate a more nuanced understanding of how food safety risks and livelihood benefits are distributed within the game meat value chain.

## 2. Materials and Methods

### 2.1. Study Site

**Figure 1:**
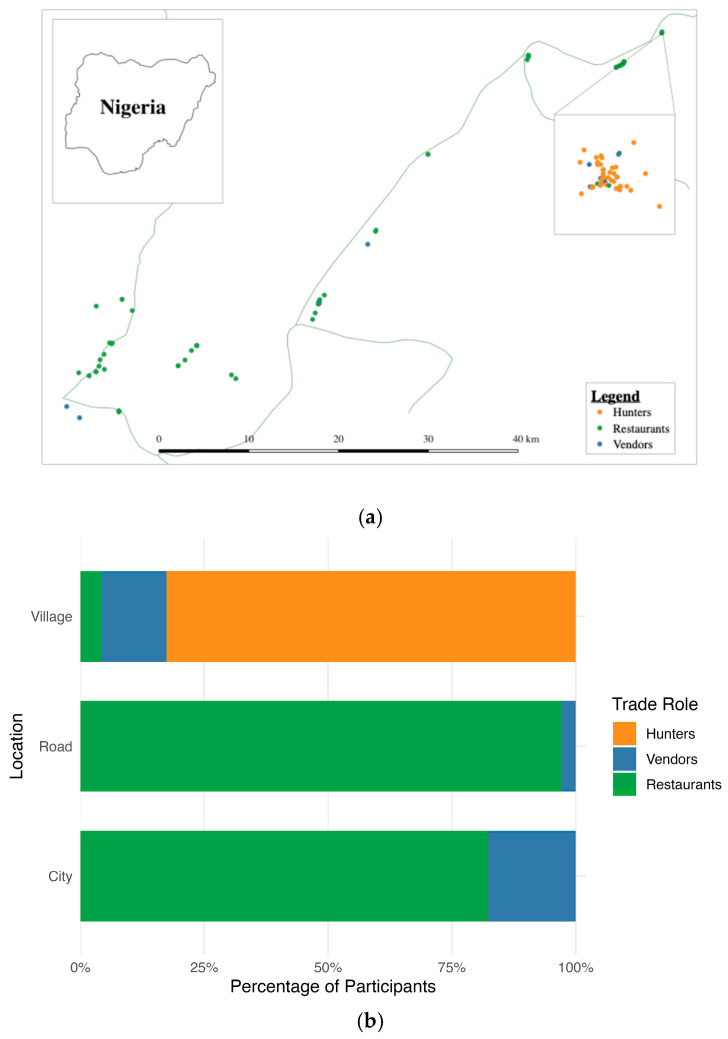
Spatial distribution of game meat trade participants and role composition by location type. (**a**) Relative spatial distribution of study participants (with hunters in orange, vendors in blue and restaurants in green) in relation to Cross River National Park and an urban center endpoint within Cross River State, Nigeria. Identifiable land features, urban structures, map orientation have been removed to protect participants’ privacy. (**b**) Stacked bar chart showing the proportional composition of trade roles within each location type (city, road, village).

The study took place in Cross River State, Nigeria and included game meat traders located (1) in a focal study village within the interior of Cross River National Park, (2) along a major roadway (~60 km) connecting rural villages to an urban center, and (3) within a major urban center (157 sq mi and over 500,000 people); see Figure 1a. Major ethnicities in this state include Efik, Ejagham, and Bekwarra people groups, with Efik the most prevalent state language beyond English [37,38]. Cross River National Park (CRNP) is a tropical rainforest characterized by dense lowland and montane forests that harbor high levels of biodiversity [39,40]. It is home to at least 75 species of mammals, 282 birds, and over 42 species of reptiles [41]. Game meat in this region is important for both household level food security, trade, and medicinal use [42,43,44].

### 2.2. Ethics Statement

This study was conducted in accordance with the Declaration of Helsinki and received ethical approval from the Institutional Review Board of The Pennsylvania State University (IRB #00019476). Additional research approvals were obtained from the Cross River State Ministry of Health (CRSMOH/HRP/REC/2023/341), the Health Research Ethics Committee of the University of Calabar Teaching Hospital (UCTH/HREC/008/67), the Conservator of Cross River National Park, and the Forestry Commission of Cross River State. Furthermore, the study was introduced to village heads and local authorities to request permissions and feedback. Similar sensitization was carried out individually with selected roadside and city traders. All participants were read a summary of the study and its benefits, risks, and protections, and provided oral consent. Written signatures were not obtained due to low literacy rates and the illegal nature of game meat trading.

### 2.3. Participants

We began enrollment activities in 2020, during which 220 prospective participants were identified. Our Nigerian field team, including two postgraduate students in Forestry and Wildlife Resources Management at the University of Calabar, visited actors within the game meat supply network ranging from hunters in remote villages to restaurants and bars along major trade routes and formal city markets. During scoping visits, our team gauged whether individuals and establishments were actively trading game meat and asked to be referred to others engaged in similar activities within their locality, a snowball sampling approach (e.g., Palinkas et al., 2015 [45]). From these scoping activities, we identified a total of 201 participants who were consented and enrolled: 61 village households, 19 of which contained self-identified hunters (individuals directly engaged in capturing wild animals), 17 middlemen and vendors (those who purchase meat for resale, inside and outside of market settings), and 123 restaurants and bars (fixed-location eateries serving game meat, often with other food items and beverages). We selected a random subset of restaurants along the roadway as this role in this location was overrepresented nearly three-fold. Ultimately, our sample comprised 91 traders, including: 19 (21%) hunters, 62 (68%) restaurants, 10 (11%) vendors across our three study locations (Figure 1b).

### 2.4. Questionnaires

Between January 2023 and April 2024, we opportunistically conducted structured interviews as part of a larger year-long study (Thompson et al. 2026 [46], in preparation). All survey instruments were initially written in English and then administered in Nigerian Pidgin English, Ibibio, and Ejagham by the survey team. We administered two survey modules; the first was a baseline survey with a one-year recall window. In this survey, we collected information to identify sociodemographic and economic variables that may broadly characterize different trade roles. This included ethnicity, and highest level of education completed, age, gender, household demographics, asset ownership, income from game meat, as well as their general beliefs and behaviors around meat handling. We also documented whether participants consumed the game meat they acquired through hunting or purchasing and/or redistributed it to other trade roles (see Supplementary Materials). We simultaneously conducted a second module that entailed a repeated-measures monthly questionnaire with a 7-day recall of wild meat interactions, documentation of how the meat was processed or preserved, and the price at which it was sold, among other variables. Species were standardized to the lowest reliable taxonomic level using multilingual photo guides to support accurate identification.

### 2.5. Variable Descriptions and Analysis

#### 2.5.1. Game Meat Handling Practices

To characterize how game meat moves through the local value chain, we asked participants from whom they acquire bushmeat and to whom that meat subsequently goes. Acquisition categories included self-procured (e.g., hunted or trapped), or sourced directly from a hunter, vendor (mobile, roadside, or market) or restaurant. Destination categories included consumers (self or other), hunters, vendors, and restaurants. We aggregated reported acquisition–destination pairs into a directed, weighted trade-flow table and visualized the resulting network as a node-link diagram using Graphviz (DiagrammeR), with nodes representing standardized actor categories and directed edges labeled by the number of reported transfers. Beyond the sociodemographic data detailed above, we asked which household members regularly handled meat. Differences in the percentage of household members engaged in the trade across trade roles were then assessed using a Kruskal–Wallis rank-sum test.

**Figure 2:**
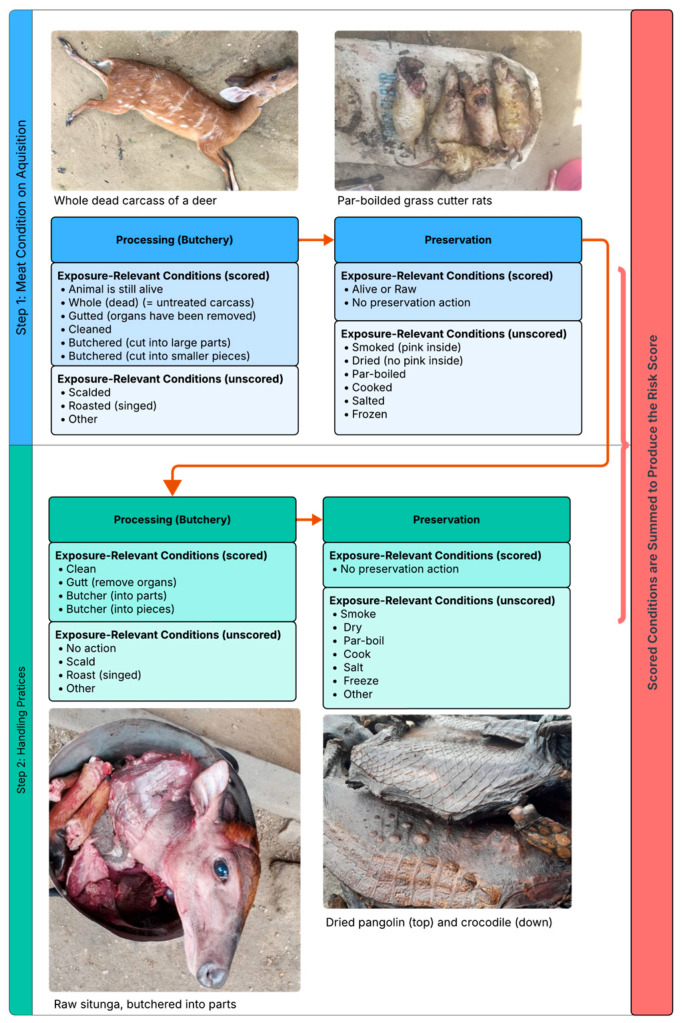
Conceptual framework illustrating how exposure-relevant conditions are classified and scored across acquisition and post-acquisition stages of game meat handling, including processing and preservation pathways used to construct the cumulative risk index.

Because trade role and associated working contexts are likely to shape how participants encounter and modify meat within the supply chain, we conceptualized meat handling as a staged pathway (Figure 2).

The first point of a participant’s contact with a carcass or meat reflects the acquisition state defined by (1) processing condition (e.g., live animal, whole carcass, gutted, butchered into parts or pieces, scalded or roasted) and (2) preservation state (e.g., raw, smoked (may be pink inside), dried (no longer pink inside), par-boiled, cooked, salted or frozen). Although participants reported ‘alive’ as an acquisition state in both processing and preservation categories, it was coded under processing only and not preservation to avoid double counting of exposure states. Further handling practices included additional processing and/or preservation action. These sequential stages created cumulative opportunities for contact with animal carcasses and meat. Participant responses were subsequently scored based on the meat conditions and handling practices that facilitated exposure to and manipulation of under preserved meat (Table 1). The resulting Risky Handling Practices Index ranged from 0–14, reflecting reported acquisition states, including: live animals (0/1), whole carcasses (0/1), gutted or manually butchered carcasses (0/3), and raw meat (0/1); and handling practices involving gutting (0/1, cutting (0/1), or cleaning (0/1), or “other “handling practices (e.g., pinning with sticks or tithing (sic) with rope) (0/1); or absence of preservation when meat as meat was kept raw (and so prolonging contact with animal bodily fluids) (0/1). Conditions involving heat treatment or preservation methods associated with reduced microbial load (e.g., cooking, smoking, drying, salting, freezing) were scored as (0) [47]. The total risky practices score ranges from 0–14, reflecting the sum of four component indices with differing numbers of contributing indicators.

We analyzed both stage-specific and overall risky practice counts using Poisson generalized linear models with trade role as the predictor. Model fit and coefficient significance were evaluated using Wald z statistics, and likelihood ratio tests were conducted to assess overall model effects.

We used average mean monthly handling time (i.e., reported number of hours a piece of meat spent in a participant’s possession) as a measure of exposure across trade roles, calculated from our repeated measures survey. For each participant, we summed reported handling hours within each person-month and then averaged across all observed person-months, ensuring that individuals sampled more frequently did not contribute disproportionate weight. We then used Kruskal–Wallis tests with Dunn pairwise comparisons (Holm-adjusted *p*-values) to compare distributions across trade roles.

We also examined injuries during handling as exposure risk factors. Injury data were collected through self-reports of both injury type (e.g., cut, puncture, scratch) and severity (mild or moderate), then recoded into a binary occurrence variable and averaged per person. To compare injury rates across trade roles, we calculated injuries per 100 handling hours, adapted to mirror standard occupational injury rate metrics [48,49]. Given the zero-inflated, skewed distribution of the data, we used Kruskal–Wallis tests with Dunn pairwise comparisons (Holm-adjusted *p*-values).

#### 2.5.2. Precautions

To understand precautionary behaviors during game meat handling and consumption, we inventoried nineteen common sanitary actions using five-point Likert-type frequency scales. Specifically, we asked participants how often they washed their hands before and after handling animals or meat (and whether they used soap), used personal protective equipment, changed their clothes after handling meat, washed the surfaces and tools used to process or prepare meat, used those surfaces and tools for other purposes. We also asked how often meat was cooked until it was no longer pink or red inside or consumed in a raw or undercooked condition. Likert items related to risky behaviors were reverse scored. We calculated a composite precaution score by averaging across behaviors, with higher scores reflecting more frequent precautionary behavior.

To assess participants’ perceptions of zoonotic disease risk associated with handling and consumption of game meat, we evaluated their awareness, beliefs, level of concern, and perceived route of transmission (e.g., consumption or butchering). We employed binomial and ordinal logistic regression models to test whether trade role predicted awareness and belief, respectively. For those who reported awareness, we used Spearman’s rank correlations to assess the association between concerns about transmission and belief in spillover risks. Perceived transmission routes were categorized and summarized descriptively as frequencies and percentages across the full sample.

#### 2.5.3. Income

To assess economic benefit across trade roles, we asked participants about their monthly average income from game meat and whether it represented their primary income source. For a more refined estimate, we drew on a subset of the monthly survey data (Thompson et al., 2026 [46], in preparation). From these data, we summed reported sale prices per person-month and then averaged across all observed months to standardize for sampling frequency. Differences in mean monthly income across trade roles were evaluated using nonparametric rank-based tests with Holm-adjusted pairwise comparisons.

To assess economic dependence and diversification, we used generalized linear models for binary and count outcomes and a nonparametric test to compare the number of secondary occupations across trade roles. All currency values are presented in Nigerian Naira (₦) and the USD equivalents in 2024.

To assess how wealth varied across trade roles, we inventoried access to utilities and livestock and durable goods. We adapted the Demographic and Health Surveys (DHS) wealth index to account for local cultural context (see Supplementary Material); water sources were categorized as improved or unimproved following DHS guidelines [50]. We compared composite wealth scores across trade roles using Poisson generalized linear models.

Trade role and location (city, road, or village) were significantly confounded (Fishers exact test; *p* < 2.2 × 10^−16^), and we therefore excluded trade location from subsequent models. Given uneven sample sizes across trade roles, we used nonparametric statistical methods throughout. All analyses were performed using R version 4.3.2 [51].

## 3. Results

### 3.1. Sociodemographics

**Figure 3:**
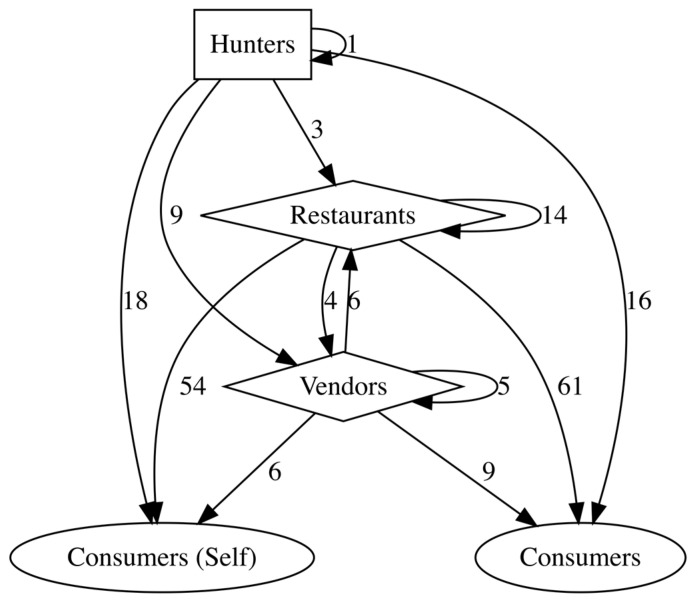
Structure of the game meat trade network in Cross River State, Nigeria.

Trade roles varied by location: hunters were clustered in the village (19/19); restaurants were more common along the road and in the city (33/62 and 28/62, respectively); and vendors were split across sites (6/10 city, 1/10 road, 3/10 village). Game meat trade circulated across trade roles in a complex, non-linear network, with bi-directional flows between hunters, vendors, restaurants, and consumers (Figure 3). Self-consumption was documented across roles, reflecting the dual economic and subsistence value of game meat to actors.

Arrows represent flows of game meat between trade roles, based on participant accounts acquisition, sales, and self-consumption. Numbers indicate the count of participants engaged in each trade pathway. Self-loops indicate meat retained for consumption within a household.

Ethnic composition differed across trade roles. Hunters were predominantly Ejagham (n = 18, 94.74%), and vendors were also predominantly Ejagham (n = 5, 50%), whereas restaurant owners were most commonly Ibibio (n = 28, 45.16%). Restaurants were the most ethnically diverse group, with seven ethnic groups represented, compared to hunters (2) and vendors (4). While both men and women engaged in the game meat trade, hunters were exclusively male. Women comprised the majority of restaurant work and vendor roles, with female-to-male ratios of 2.33 and 4.64, respectively (Table 2). Though household size was relatively similar across trade roles, the percentage of household members engaged in the trade varied significantly (χ^2^ = 12.94, df = 2, *p* = 0.002), with restaurants reporting the highest mean percentage of household members engaged in the trade (67.31%). Across roles, the most common supplementary livelihoods were farming, trading other goods, and harvesting non-timber forest products. All hunters reported farming (n = 19, 100%). Restaurants most commonly reported farming and trading goods (n = 19, 30.6% for each), while vendors most commonly reported farming (n = 5, 50%) and trading goods (n = 4, 40%). Vendors reported the highest overall average monthly income (₦45,884; n = 10), followed by restaurants (₦36,860 ± ₦8219 SE; n = 62) and hunters (₦25,086 ± ₦4812 SE; n = 19).

### 3.2. Game Meat Handling Practices

A person’s trade role significantly affected how frequently they interacted with risky meat conditions, evidenced by significantly different total risky practices scores (χ^2^ = 20.54, df = 2, *p* < 0.001). Overall, hunters handled meat in high-risk states 50.90% more often than restaurants and 75.80% more often than vendors.

**Figure 4:**
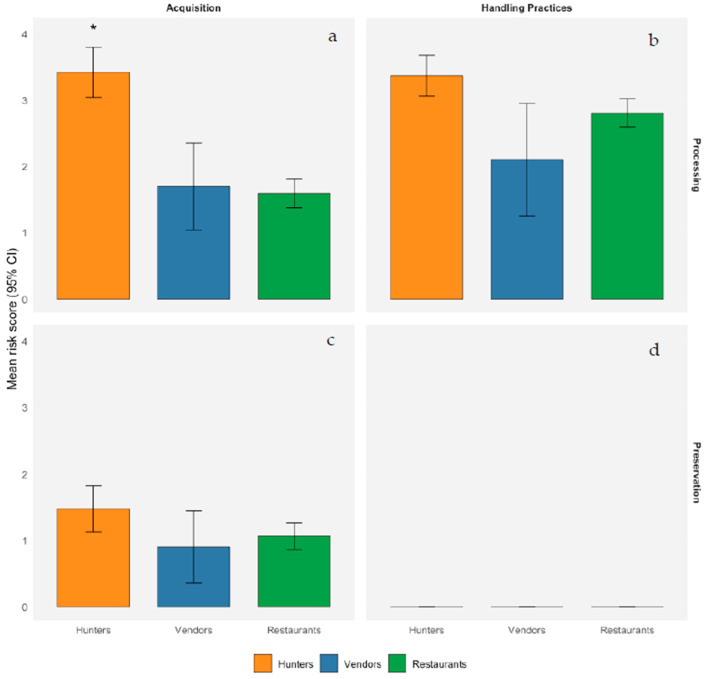
Mean risk scores (±95% confidence intervals) for meat handling practices by trade role across stages of interaction. Panels are organized vertically by stage (Acquisition and Handling Practices) and horizontally by step (Processing and Preservation). Significant differences (*) among trade roles were observed for processing-related risk at acquisition, with hunters exhibiting the highest mean risk scores.

Risk scores also varied by stage of meat preparation (Figure 4). The processing state of meat upon acquisition differed significantly between trade roles (χ^2^ = 21.67, df = 2, *p* < 0.001), with hunters exhibiting the highest mean risk count (3.42, SE = 0.19), followed by restaurants (53.30% reduction; *p* < 0.001), and vendors (50.30% reduction; *p* = 0.01) (Figure 4a). Trade roles did not significantly differ in handling practices (*p* > 0.05 for all processing and preservation scores) (Figure 4b,c). Although preservation risk scores did not differ statistically, preservation methods varied across roles: hunters primarily relied on drying and cooking, whereas restaurants also reported freezing meat. Vendors most commonly relied on drying and smoking.

**Figure 5:**
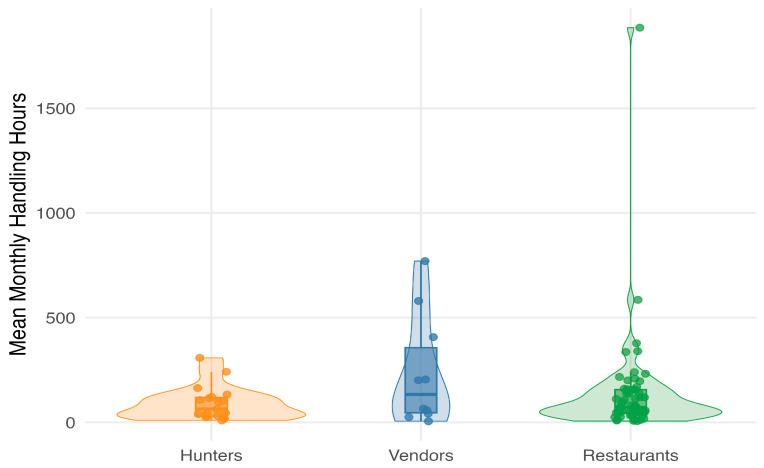
Distribution of individual mean monthly handling exposure across roles. Each point represents one individual’s mean monthly handling time (measured as hours in a participant’s possession), violin plots show the distribution of individual values within each role, and boxplots indicate the median and interquartile range.

Across individuals, mean monthly handling time was 90.30 h for hunters (n = 19), 139.00 h for restaurants (n = 60), and 236.00 h for vendors (n = 10); (Figure 5). Differences in individual mean monthly handling exposure were not statistically significant (χ^2^(2) = 1.73, *p* = 0.42). Pairwise adjusted *p*-values ranged from 0.58 to 0.80, and rank-based effect sizes were small (r = 0.10 to 0.22).

Total reported injuries per individual were low across all trade roles, ranging from 0–6 among restaurant owners (mean = 0.32, SE = 0.14), 0–1 among vendors (mean = 0.10, SE = 0.10), and 0–1 among hunters (mean = 0.05, SE = 0.05). When standardized by handling time, mean injury rates per 100 handling hours were 0.13 ± 0.07 for restaurants, 0.01 ± 0.01 for hunters, and negligible for vendors. Injury rates did not differ significantly across trade roles (χ^2^(2) = 0.67, *p* = 0.72).

### 3.3. Precautions

Trade role significantly influenced the frequency of hygienic precautions were adopted during meat processing and preparation (χ^2^(2) = 42.04, *p* < 0.001). Restaurant workers reported the highest frequency of precautionary behaviors (mean = 4.15, SE = 0.05), followed by vendors (mean = 3.61, SE = 0.22), while hunters reported the lowest scores (mean = 3.13, SE = 0.08), with pairwise differences significant after Holms’s correction.

**Figure 6:**
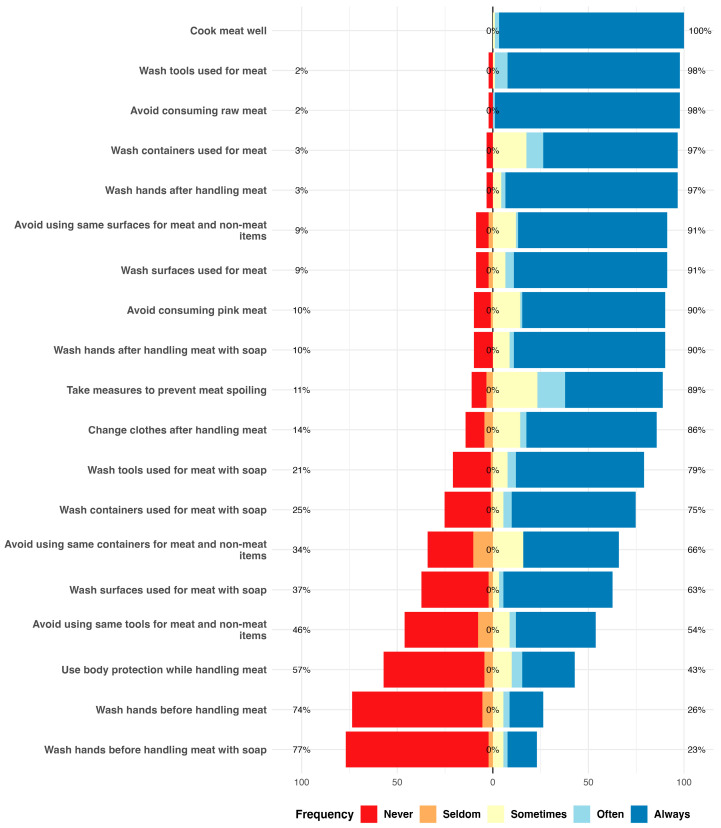
Distribution of reported hygiene practices among game meat handlers. Bars show the percentage of participants selecting each Likert response category (Never [red] to Always [blue]) for each hygiene behavior. Responses are centered at neutral to display divergence between lower-frequency and higher-frequency behaviors.

While most participants reported always cooking meat thoroughly (96.70%, n = 88) and avoiding consumption of raw (96.70%, n = 88) or undercooked (pink) meat (74.73%, n = 68), hand hygiene was notably poor—68.13% (n = 62) reported never washing their hands before handling meat, and 74.73% (n = 68) never doing so with soap (Figure 6).

Role differences were most pronounced for washing surfaces, containers, and tools (with and without soap), changing clothes and washing hands after handling meat, use of personal protective equipment, spoilage protection measures (all χ^2^ tests *p* < 0.05; Supplementary Table S1). Restaurant workers consistently reported higher adoption of protective behaviors than hunters, with vendors intermediate (Supplementary Table S2). No significant role differences were detected for hand washing prior to handling meat (with or without soap) or avoidance of shared surfaces, tools, and raw/undercooked meat (all *p* > 0.05; Supplementary Table S1).

Most participants reported awareness of zoonotic diseases (80.00%, n = 72), though only 25.00% (n = 18) of those reporting awareness agreed or strongly agreed that personal zoonotic disease risk existed. Awareness and belief did not differ significantly across trade roles (all *p* > 0.05). Among those who were aware, stronger belief in zoonotic disease (coded as lower values) was associated with greater concern over infection (ρ = −0.57, *p* < 0.001). When asked about transmission routes, over half of participants indicated that zoonotic transmission does not occur (54.44%, n = 49/90). Among those who named a transmission route (14.44%, n = 13), consumption of animal products (15.56%, n = 14) and direct contact during processing, cooking (14.44%, n = 13), or hunting (11.11%, n = 10) were most commonly cites.

### 3.4. Income

Income differed significantly across trade roles (χ^2^(2) = 20.10, *p* < 0.001). Mean monthly income from game meat was ₦20,338 (≈ $32; SE = ₦3162) for hunters (n = 18), ₦65,669 (≈ $104; SE = ₦8233) for restaurants (n = 59), and ₦138,135 (≈ $218; SE = ₦43,626) for vendors (n = 10). Hunters earned significantly less than both restaurants and vendors (moderate effects, r = 0.45 and 0.38 respectively), while restaurants and vendors did not differ significantly (r = 0.08; *p* = 0.69).

**Figure 7:**
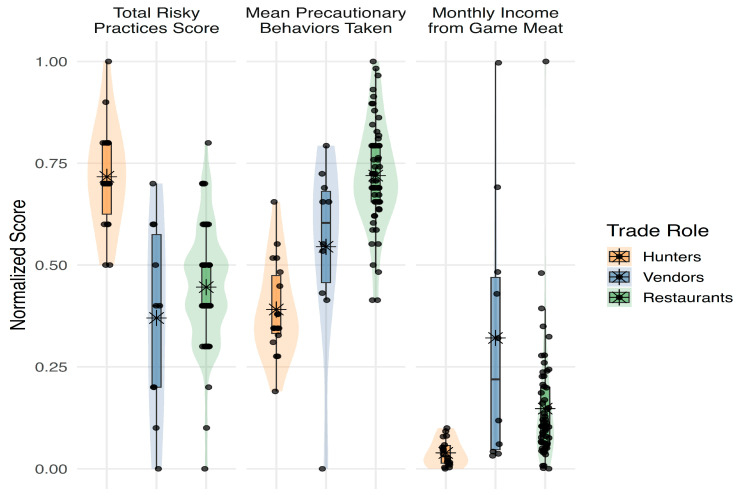
Normalized distributions of income, risky practice exposure, and precautionary behavior across game meat trade roles. Min-max normalized distributions of monthly income from game meat, total risky practices score, and mean precautionary behavior frequencies, shown as violin plots with embedded boxplots for hunters (orange), restaurants (green), and vendors (blue). Asterisks indicate group means. All values scaled 0–1 to enable cross-metric comparison.

Results across exposure, precautionary behavior, and income are synthesized in Figure 7.

Game meat was the primary income source for most vendors (60.00%; n = 6) and restaurant workers (51.61%; n = 32), but rarely for hunters (5.26%; n = 1) (both *p* < 0.01). Hunters also reported the greatest number of alternative income streams outside the game meat trade (mean = 2.37, SE = 0.19), compared to restaurants (mean = 1.61, SE = 0.10) and vendors (mean = 1.40, SE = 0.37) (χ^2^(2) = 12.81, *p* = 0.0017), with farming and trading other goods the predominant supplementary livelihoods among all roles. Wealth differed significantly across trade roles (χ^2^ = 4044.23, *p* < 0.001). Hunters had significantly lower wealth index scores than both restaurants and vendors (all *p* < 0.01), while no significant difference was observed between restaurants and vendors (*p* = 0.68).

## 4. Discussion

The game meat supply chain in Cross River State, Nigeria appears to concentrate occupational risk and economic vulnerability among the same actors. Hunters, who handle meat in risky conditions most frequently, adopt the fewest precautionary measures, and earn the lowest income from game meat (Figure 7), may be positioned within the trade in ways that systematically expose them to greater risk while limiting economic return. This pattern suggests that the organization of the game meat trade itself may produce uneven distributions of both health risk and livelihood benefit, rather than these differences arising solely from individual behavior or choice. The network is also more complex than often assumed [52], with unequal populations across trade roles and an overall nonlinear structure. This has implications for how interventions should be designed and targeted. Additionally, while awareness of zoonotic disease was high, personal concern was universally low across trade roles, suggesting that information alone is unlikely to drive behavior change.

### 4.1. Zoonotic Exposure Risk

Our results reinforce the notion that hunters face the greatest occupational zoonotic exposure risk within the game meat supply chain, consistent with their role as primary slaughterers of fresh carcasses and the frequency with which they contact blood and viscera [2,4,53]. However, our results also demonstrate that risk does not end with hunters. Restaurant workers and vendors—often considered “downstream” in the supply chain and therefore assumed to be buffered from these risks—still regularly handle raw and undercooked meat. Butchery, preservation, and preparation activities were reported across all trade roles, rather than being confined to a single stage of the supply chain. Handling time did not differ significantly across roles, suggesting that duration of contact with meat is broadly similar across actors despite differences in role. As such, risk is not confined to the point of harvest but is distributed across the supply chain.

Although overall injury rates were low and not statistically significant, restaurant workers in our study remained the most likely to injure themselves during handling. This mirrors injury patterns among workers in formal restaurant settings [54]. Yet within the literature on game meat, explorations of injury-related risks are often confined to hunters [55], and remain largely unquantified. Our findings suggest that this focus on hunters may overlook downstream actors in the trade, particularly women working in restaurant and vendor roles that entail frequent meat handling [21,56]. Furthermore, while restaurant workers are commonly viewed as the source of foodborne illness outbreaks that pose risk to consumers [57], these workers remain understudied as subjects of occupational risk despite frequent exposure to meat that may carry bacterial, viral, and parasitic pathogens [58].

While exposure risk appears more distributed across the supply chain than often described in the literature, precautionary behaviors were unevenly adopted, with lower engagement among hunters relative to other trade roles. Hunters’ lower uptake of hygienic behaviors may reflect their working environments: often located far from urban infrastructure, grid utilities, and access to market goods such as soap and protective equipment. These constraints may contribute to less frequent sanitizing of hands, containers, and surfaces, as well as reduced use of protective clothing. Similar behavioral patterns among hunters have also been documented in game meat trade chains in the Democratic Republic of Congo [59]. Low adoption of soap and personal protection equipment overall is consistent with other observations in other Nigerian contexts [60,61] and elsewhere [62]. However, even where these amenities are available, the adoption of hygienic practices may remain low if individuals do not perceive meat handling to be inherently risky [22,63]. While game meat traders in urban locales may be better equipped to take preventative measures, risk mitigation campaigns are not effective if risk is not perceived [64,65]. We found that while awareness of zoonotic disease was high (80%), fear of exposure was universally low regardless of trade role (25%), and this level of concern did not increase with personal experience with sick animals. Given this, public campaigns may be effective in raising awareness, but less effective in raising the feelings of concern needed to motivate individuals to modify their behavior [16].

In our study, restaurants did report the most frequent participation in hygienic behaviors, potentially because traders situated in urban locations may have greater access to running water via public utilities, as well as market goods such as manufactured cleaning products and personal protective equipment [60,61]. This pattern suggests that access to infrastructure and resources, rather than risk awareness alone, may shape the adoption of precautionary behaviors across trade roles [66,67,68]. Similar combinations of constraints have been linked to reduced uptake of food safety practices in other resource-limited settings. For example, in poultry restaurants in Burkina Faso, tools and clothes were commonly reused without cleaning between processing and food preparation, and meat was often kept at room temperature despite access to refrigeration [66]. In this context, most staff lacked formal training on food handling and felt cleanliness was not important to customers, which may have correlated to low uptake of protective practices. These challenges are hardly unique to low- and middle-income countries, or the game meat trade more specifically; microbial foodborne illnesses involving meat, often resulting from failures to adhere to food safety regulations, remain a persistent problem in the United States and Europe as well [69,70]. However, unlike legally-sanctioned and institutionally-regulated supply chains, criminalized game meat industries may entail higher hygiene-related health risks because they function cryptically [71]. The game meat trade may benefit from destigmatization in order to facilitate the normalization of hygienic practices. This can be combined with context-specific solutions—potentially drawing from best practices in field dressing meat [72] and food safety in such as wilderness contexts [73]. Such approach account for, rather than ignore, constraints in access to clean water and personal protective equipment.

### 4.2. Benefit

The wide variability of income from game meat suggested inequitable gains between participants. Income varied substantially, with vendors in particular generating the highest average revenue. These traders also exhibit the highest relative incomes in other parts of Nigeria where their intermediate position (i.e., middlemen) allows them to purchase directly from hunters at relatively low prices and reselling to end-point consumers at higher profits [74]. This aligns with broader evidence that value chains for wild meat are structurally unequal, with gains distributed unevenly across roles. In central Amazonia, for example, reported sale prices rose from R$1.50 to 2.00/kg in rural villages to R$5.50/kg in urban markets, indicating a substantial downstream price gradient [75]. Carcass prices similarly increased between hunters in source locations and resellers in urban areas in Gabon [76]. However, higher downstream sale prices do not necessarily translate into higher relative profitability for downstream actors. In Tanzania, hunters showed higher cost-benefit ratios than consumer-facing retailers, suggesting that upstream actors sometimes have stronger returns relative to investment costs even when end-market prices are ultimately higher farther along the chain [16,77]. While we did not observe a clear linear increase in income from hunters to vendors to restaurants, geographic variation in income was still pronounced, with the highest earners located in urban areas and the lowest residing in the focal village.

Although hunters earned the least from the trade on average, this income may nonetheless be extremely important for their livelihoods. Hunters exhibited lower baseline wealth, more reliance on natural resource-based occupations, and fewer alternative income opportunities due to their remote location. Future qualitative research should therefore explore not only the quantity of earnings generated through bushmeat hunting, but also its timing, perceived value, and usage within household budgets. As different people depend on game meat to different degrees, legal bans and species loss will not affect all actors equally [78]. Livelihoods dependent on game meat stand to be destabilized by decreased access to wildlife (either due to species extirpation, heightened conservation enforcement, or public health interventions restricting the trade). Recently, concern about zoonotic disease spillover has intensified prohibitive legislation in many countries, including as Nigeria [79]. Such tightened regulations may jeopardize income and food security for actors who already lack the socioeconomic capital required to pivot livelihood strategies [80]. This is especially concerning where alternative employment opportunities are limited [81]. As such, equitable solutions should be developed using participatory frameworks that consider the structural economic inequality inherent to the game meat trade.

### 4.3. Solutions

Our findings suggest that the game meat trade can be understood through an occupational health lens rather than through exceptionalism or exoticism. The patterns we document (specifically role-stratified risk due to variation in the condition of meat handled, handling norms, and hygienic practices) mirror dynamics in other occupations that involve frequent human-wildlife interactions. In laboratory and zoo contexts, for example, exposure is driven by tasks with high contact intensity shaped by workers’ perceptions of their own vulnerability [82,83]. Similarly, work from agricultural contexts suggest social constructs influence workers’ adoption of preventive behaviors [84]. Yet such occupational insights have rarely been applied to game meat handling; there risk mitigation strategies could be adapted to the cultural context, community priorities, and belief systems embedded in the wildlife trade [85]. Framing the game meat sector as just one of many high-animal-contact occupations facilitates the application of standardized food safety and occupational health frameworks, which may improve the uptake of protective behaviors [86].

One such approach, the Social Ecology of Occupational Zoonoses (SEOZ) framework, integrates (1) individual-level behavioral and cognitive determinants (e.g., risk perception, self-efficacy), (2) interpersonal and organizational dynamics (e.g., peer norms, safety culture, management practices), and (3) environmental and structural conditions (e.g., workplace context, exposure pathways) to inform mitigation practices at multiple scales [84]. This approach that not only examines the hazardous actions taken by workers, but crucially considers worker’s comprehension of the consequences alongside their ability to prevent them [87]. Studies on safety within the game meat trade could consider the role of personal agency (or one’s sense of one’s own ability to affect change) [88] in buffering against risks. This may be done through the formal application of capability-opportunity-motivation frameworks common to behavioral psychology [89]. Furthermore, future research would benefit from risk assessment tools that integrate worker behavior, self-perception, and network dynamics with microbial hazard data. This would allow for behavioral, contextual, and biological sources of risk to be evaluated together rather than in isolation. Such tools could help identify where unsafe practices coincide with the greatest pathogen burden, improving surveillance and intervention.

## 5. Conclusions

Viewing the game meat trade as a nonlinear supply chain comprised of distinct workplace roles reveals uneven role-specific hazards and variable economic returns. Hunters encounter the most frequent high-risk meat contact and take the fewest precautions while earning the least, whereas vendors and restaurants receive more income from the trade. Taken together, these findings show that game meat supply chains do not merely distribute food and income but actively produce inequities by concentrating zoonotic risk among the least economically protected actors. Low belief in disease risk and uneven access to amenities such as protective equipment, plumbing, and cold storage may further contribute to these vulnerabilities. However, this study is not intended to be representative of all of Nigeria, and future work should examine game meat trade networks across culturally and ecologically diverse settings.

Given this, broad awareness campaigns alone are likely insufficient to affect change. Overall, the impact of top-down behavioral interventions in the game meat trade ultimately depends on how they shift risks relative to benefits among diverse actors. We recommend that occupational health interventions be co-designed with traders to reduce spillover risk without deepening poverty. Future work should position food safety and zoonotic disease prevention as ethical obligations that must be addressed at the level of supply-chain structure, rather than solely through individual behavior change.

## Figures and Tables

**Table 1:** Definition and scoring of exposure-relevant conditions across acquisition and handling stages in the game meat value chain.

Stage	Stage	Score Range	Exposure-Relevant Conditions Counted in the Score
Acquisition State	Processing Condition	0–7	Live animal at acquisition (1); whole dead (1); gutted carcass (1); cleaned carcass (1); butchered into parts (1); butchered into pieces (1); other (1)
	Preservation Condition	0–2	Raw at acquisition (1); no preservation action at acquisition (1)
Handling Practices	Processing Actions	0–4	Gutting (1); cleaning (1); butchering into parts (1); butchering into pieces (1)
	Preservation Actions	0–1	No preservation action following acquisition (1)
Overall Index	Total	0–14	Sum of all exposure-relevant conditions across the four stages

**Table 2:** Sociodemographic summaries of participants; all variance indicated as standard error.

Description	Hunters	Vendors	Restaurants
Sample Size	19	10	62
Female: Male Ratio	0	2.33	4.64
Average age	45.16 ± 2.8	36.6 ± 3.49	38.65 ± 1.88
Family size	5.32 ± 0.48	5.6 ± 1.1	5.65 ± 0.37
% of Household Members Engaged in Trade	43.02	46.4	67.31
Education beyond primary school (%)	89.47	70	72.58
Occupation (%)—Farmer	100	50	30.65
Occupation (%)—Harvests non-timber forest products	21.05	20	3.23
Occupation (%)—Trade goods	0	40	30.65
Average Monthly Income (₦)	25,086 ± 4812	45,884 ± 17,432	36,860 ± 8219

## Data Availability

Data are available upon reasonable request.

## Supplementary Materials

The following supporting information can be downloaded at: https://www.mdpi.com/article/10.3390/ani16111666/s1.
