# Supplementary material for: Occupational Exposure and Economic Inequity Along a Game Meat Trade Network in Cross River State, Nigeria
Source: Animals (Basel). 2026 May 29;16(11):1666. doi: 10.3390/ani16111666 (PMC13255818; doi:10.3390/ani16111666)
Supplement: Supplementary file 1 [file animals-16-01666-s001.zip › animals-4263378-supplementary.pdf]

animals

MDPI

Supplementary Materials: Surveys

Supplementary Materials S1: Annual Survey

HOUSEHOLD/BUSINESS INFORMATION (warm-up questions)	
Ethnicity	Ejagham<br>Ibibio<br>Efik<br>Igbo<br>Other
Nationality	
What is your occupation?<br>*Select multiple.*	Farming<br>Hunting or trapping<br>Fishing<br>Timber<br>Collecting forest goods (NTFPs)<br>Animal husbandry<br>Trading goods<br>Skilled trade<br>Driver<br>Teacher<br>Student<br>Government worker<br>Other
Within the last year, you or anyone in your household interacted with bush animals in any of the following ways: *Note: read the responses to interviewees*<br>*Select multiple.*	Haven't interacted with bush animals<br>Caught bush animals<br>Carried meat<br>Purchased meat<br>Sold meat<br>Processed meat (butchered or gutted)<br>Preserved meat (singed, smoked, dried, or cooked)<br>Ate meat<br>Played with bush animals (live)<br>Played with bush animals (dead)<br>Played with bush meat<br>Cared for live bush animals<br>Medicine<br>Tools/cultural use<br>Other
Which species did you specifically interact with?	None

https://doi.org/10.3390/ani16111666

Select multiple.	[SPECIES LIST]<br>Other
Which species, if any, have you avoided touching?<br>*Select multiple.*	None<br>[SPECIES LIST]<br>Other
Which species, if any, have you avoided consuming?<br>*Select multiple.*	None<br>[SPECIES LIST]<br>Other
Please list all the places where you process your bush-meat?	Hunting shed<br>Household kitchen<br>Area around house/ compound<br>Shop or restaurant kitchen<br>Area around shop/ restaurant<br>Market stall<br>Other
Where is the primary place that you process your bush-meat?<br>*Skip logic: If interaction = !Haven't*	Hunting shed<br>Household kitchen<br>Area around house/ compound<br>Shop or restaurant kitchen<br>Area around shop/ restaurant<br>Market stall<br>Other
Please list all the places where you preserve your bush-meat?<br>*Skip logic: If interaction = !Haven't	Hunting shed<br>Household kitchen<br>Area around house/ compound<br>Shop or restaurant kitchen<br>Area around shop/ restaurant<br>Market stall<br>Other
Where is the primary place that you preserve your bushmeat?<br>*Skip logic: If interaction = !Haven't*	Hunting shed<br>Household kitchen<br>Area around house/ compound<br>Shop or restaurant kitchen<br>Area around shop/ restaurant<br>Market stall<br>Other
What is the main source of water for cooking, cleaning and handwashing in the place where you process the majority of your bush meat.<br>*Select one*<br>*Skip logic: If interaction = !Haven't	Piped water: into building<br>Piped water: yard/plot<br>Piped water: to neighbor<br>Piped water: tap/standpipe<br>Tube well or borehole<br>Dug well: protected

https://doi.org/10.3390/ani16111666

	Dug well: unprotected<br>Water from spring: protected<br>Water from spring: unprotected<br>Rainwater<br>Tanker truck<br>Cart with small tank<br>Surface water (river/dam/lake/pond/stream/canal/irrigation channel)<br>Bottled water<br>Sachet water<br>Other
Where is that water source located?<br>*Select one.*	In own house<br>In own compound (outside house)<br>Elsewhere<br>Other
About how long does it take to get water and come back (if not available on site)?	(# minutes)
Do you do anything to the water to make it safer to drink or use?<br>*Select multiple.*	No<br>Boil<br>Add bleach/chlorine<br>Strain through a cloth<br>Use water filter (ceramic, sand, composite, etc.)<br>Solar disinfection<br>Let it stand and settle<br>Alum<br>Unknown/don't know<br>Other
In the past two weeks, was the water from your main source not available for at least one full day?	No<br>Yes
Including yourself, how many people live in your household?	(# people)
Say: Now we're going to ask you to tell us the gender and age of the different people in your household and how they interacted with animals within the past year.<br><br>***Let's start with you.***	
Gender	Male<br>Female
Age	(# years)
In the past year, what ways have you/ this person interacted with bushmeat?<br>*Note: read the responses to interviewees as needed*	Haven't interacted with bush animals<br>Caught bush animals<br>Carried meat

https://doi.org/10.3390/ani16111666

Select multiple.	Purchased meat<br>Sold meat<br>Processed meat (butchered or gutted)<br>Preserved meat (singed, smoked, dried, or cooked)<br>Ate meat<br>Played with bush animals (live)<br>Played with bush animals (dead)<br>Played with bush meat<br>Cared for live bush animals<br>Medicine<br>Tools/cultural use<br>Other
Does this individual assist with this business?	NA<br>No<br>Yes
Note: Repeat these questions until you have recorded information for all members of the household.	
How many people work here, excluding members of your household?<br>*This includes children.*	(# people)
Say: Now we are going to ask you about other people who do not live in your household, that help you hunt, process or prepare meat? This includes children.	
Gender	Male<br>Female
Age	(# years)
In the past year, have they interacted with bush animals in any of the following ways?<br>*Note: read the responses to interviewees as needed*<br>*Select multiple.*	Haven't interacted with bush animals<br>Caught bush animals<br>Carried meat<br>Purchased meat<br>Sold meat<br>Processed meat (butchered or gutted)<br>Preserved meat (singed, smoked, dried, or cooked)<br>Ate meat<br>Played with bush animals (live)<br>Played with bush animals (dead)<br>Played with bush meat<br>Cared for live bush animals<br>Medicine<br>Tools/cultural use<br>Other
Note: Repeat this set of questions until the respondent states there are no additional people.	

https://doi.org/10.3390/ani16111666

**SUPPLY CHAIN NETWORK**	
How do you acquire your bush meat?<br>*Select multiple.*	Household<br>Hunter (self)<br>Hunter (other)<br>Middleman<br>Vendors – Mobile<br>Vendors – Market<br>Vendors – Roadside<br>Restaurants<br>None of the above<br>Other
If hunter (other) is selected:<br>Do you sponsor (pre-pay) for meat?	No<br>Yes
What area is your bushmeat coming from?<br>*Select multiple.*	Oban Division<br>Cameroon<br>Unknown<br>Other
Do you get more bushmeat in the wet or dry season?	Wet Season<br>Dry Season<br>Both equal
Do you sell any items other than bushmeat?<br>*Select multiple.*	No<br>Cow meat<br>Pig meat<br>Chicken meat<br>Goat meat<br>Fish + Marine Animal meat<br>Dog meat<br>Palm wine<br>Agricultural foods<br>Packaged foods<br>Cooked foods<br>Non-food items<br>Other
How long has it been since most animals that you acquired were killed until they are in your possession?<br>*Hunters: how long does meat stay with you after killing but before you sell or gift?*	(# hours)
How is the meat you acquire usually **processed**?<br>*When you first get this meat, what form is it usually in?*<br>*Select multiple.*	Alive<br>Whole (dead)<br>Cleaned<br>Scalded<br>Gutted (organs removed)<br>Butchered (parts)

https://doi.org/10.3390/ani16111666

	Butchered (pieces)<br>Roasted (singed)<br>Other
How is the meat you acquire usually **preserved**?<br>*Select multiple.*	Alive<br>Raw<br>Smoked (pink inside)<br>Dried (until no pink inside)<br>Par-boilded (streamed)<br>Cooked (boiled, bbq)<br>Salted<br>Frozen<br>Other
How do you usually **process** meat that you acquire?<br>*Select multiple.*	No action<br>Clean<br>Scald<br>Gutt (remove organs)<br>Butcher (into parts)<br>Butcher (into pieces)<br>Roast (singed)<br>Other
How do you usually **preserve** that meat?<br>*Select multiple.*	No action<br>Smoke (until pink inside)<br>Dry (until no pink inside)<br>Par-boiled (steam)<br>Cook (boil, bbq)<br>Salt<br>Freeze<br>Other
Where does your meat go?<br>*Select multiple.*	Consumer (self)<br>Consumer (other)<br>Hunter (other)<br>Middlemen<br>Vendors – Mobile<br>Vendors – Market<br>Vendors – Roadside<br>Restaurants<br>Other
When you process meat, are there any parts that you always dispose of, either because it is inedible or undesirable?<br>*Select multiple.*	None<br>Bones<br>Brain<br>Heart<br>Lungs<br>Liver

https://doi.org/10.3390/ani16111666

	Spleen<br>Intestines (small or large)<br>Pancreas<br>Gallbladder ('bitter')<br>Stomach (rumen)<br>Urinary bladder<br>Kidneys<br>Male reproductive organs (testes, baculum/penis, prostate)<br>Female reproductive organs (uterus, Fallopian tubes, ovaries)<br>Hooves<br>Shell/Skin/Scales<br>Other
Aside from the parts that you always throw away, do you ever discard meat that you would normally use or sell?	Never<br>Seldom<br>Sometimes<br>Often<br>Always
If yes, why?<br>**skip logic**	Spoiled<br>Infested (by bugs)<br>Eaten by animals (cat, dog, rodent)<br>To create demand<br>Other
How much do you think you discarded last year?<br>*For each, write the number, species and unit: For example "1 whole rabbit, 5 pieces of duiker"*<br>**skip logic**	

**BARRIERS**<br>*Skip Logic: Only those who SELL*	
How difficult is it for someone new to get into this your business [of hunting, trading, or selling bushmeat (including cooked meals)]?	Easy (low start-up costs and not many challenges)<br>Moderately difficult (medium start-up costs and some challenges)<br>Difficult (high start-up costs and many challenges)
Do you have many competitors?	Low (few competitors)<br>Medium (some competitors)<br>High (many competitors)
Other than money, are there other reasons you are in the bushmeat business/market?	No<br>Prestige

https://doi.org/10.3390/ani16111666

Select multiple.	Tradition<br>Heritage<br>Relationships<br>Other
Is there anything that would make you to stop working in the bush-meat business/market?<br>*Select multiple.*	Harder to get animals<br>Lower ammunition supply<br>Higher cost of goods<br>More government oversight/regulations<br>Alternative employment<br>Nothing<br>Other

HYGIENE & SANITATION	
Now we are going to ask you some questions about your practices while handling bushmeat. Please respond by telling us if you do any of the following things, and how often (e.g., never, seldom, sometimes, often, always).	
Do you wash your hands ***before*** handling animals or meat?	Never<br>Seldom<br>Sometimes<br>Often<br>Always
If so, do you use soap?<br>*skip logic*	Never<br>Seldom<br>Sometimes<br>Often<br>Always
Do you wash your hands ***after*** handling animals or meat?	Never<br>Seldom<br>Sometimes<br>Often<br>Always
If so, do you use soap?<br>*skip logic*	Never<br>Seldom<br>Sometimes<br>Often<br>Always
Do you use any items to protect your body from contamination while you handle meat?	Never<br>Seldom<br>Sometimes<br>Often<br>Always

https://doi.org/10.3390/ani16111666

If so, what do you use?<br>**skip logic**	Gloves<br>Wrapper<br>Apron<br>Nylon<br>Overalls<br>Eye wear<br>Other
Do you change your clothes after handling meat?	Never<br>Seldom<br>Sometimes<br>Often<br>Always
Do you wash the containers or bags used for storage and transport of meat?	Never<br>Seldom<br>Sometimes<br>Often<br>Always
If so, do you use soap?<br>**skip logic**	Never<br>Seldom<br>Sometimes<br>Often<br>Always
Are those same containers or bags used to store other non-meat foods?<br>**skip logic**	Never<br>Seldom<br>Sometimes<br>Often<br>Always
Do you wash the surfaces where you process or prepare meat?	Never<br>Seldom<br>Sometimes<br>Often<br>Always
If so, do you use soap?<br>**skip logic**	Never<br>Seldom<br>Sometimes<br>Often<br>Always
Are those surfaces used to process other non-meat foods?	Never<br>Seldom<br>Sometimes<br>Often<br>Always
Do you wash the tools you use to process or prepare meat?	Never

https://doi.org/10.3390/ani16111666

	Seldom<br>Sometimes<br>Often<br>Always
If so, do you use soap?<br>*skip logic*	Never<br>Seldom<br>Sometimes<br>Often<br>Always
Are those same tools used to process other non-meat foods?	Never<br>Seldom<br>Sometimes<br>Often<br>Always
On average, how many hours do you keep meat in a raw state before you preserve it?	(# hours)
Do you use refrigeration, salting, par-boiling, or any other method to prevent the meat from spoiling?	Never<br>Seldom<br>Sometimes<br>Often<br>Always
If so, what methods do you use?<br>*skip logic*	Freezing<br>Drying<br>Smoking<br>Salting<br>Par-boiling<br>Cooking<br>Other
On average, how many hours do you keep the meat after you preserve it until it is consumed, sold, traded, or gifted?	(# hours)
Do you cook your meat until it is no longer pink or red inside?	Never<br>Seldom<br>Sometimes<br>Often<br>Always
Do you consume meat that is still pink or red inside?	Never<br>Seldom<br>Sometimes<br>Often<br>Always
Do you consume raw meat intentionally?	Never<br>Seldom<br>Sometimes

https://doi.org/10.3390/ani16111666

	Often<br>Always
Is there anything that prevents you from taking any of those protective actions that we just discussed?<br>*Select multiple.*	Expensive<br>Not available<br>Inconvenient<br>Not needed<br>Other
Do you do anything else to protect yourself when handling animals or meat?	No<br>Yes
If yes, what do you do to protect yourself when handling animals or meat?<br>**skip logic**	
Aside from any of these things we discussed, anything else you do to protect yourself from getting sick from the meat?	No<br>Yes
If yes, what do you do to protect yourself from getting sick from the meat?<br>**skip logic**	
Are there items you wish you had to protect yourself better?	Gloves<br>Wrapper<br>Apron<br>Nylon<br>Overalls<br>Eye wear<br>Other

**EXPERIENCE WITH ANIMAL SICKNESS**	
Have you ever seen a bush animal (dead or alive) that looked sick?	No<br>Yes
What species did you see?<br>**skip logic**	[SPECIES LIST]
Was this animal dead or alive?<br>**skip logic**	Dead<br>Alive
What signs made you think that it was sick?<br>*Select multiple.*<br>**skip logic**	Worms<br>Color<br>Lesions<br>Emaciated<br>Wounds<br>Endoparasites<br>Ectoparasite<br>Growths<br>Behavior

	Other
What parts of its body were affected?<br>**skip logic**	Whole<br>Hand<br>Lap<br>Trunk<br>Head + neck<br>Internal organs<br>Other
What sickness do you think it had?<br>**skip logic**	
What did you do with this animal?<br>**skip logic**	Touched<br>Consumed<br>Sold<br>Burned/Disposed/Discarded<br>Left Alone<br>Other
Do you remember seeing another sick bush animal in addition to this one?<br>**skip logic**	No<br>Yes
Repeat the set of questions by species, until the respondent says they did not see any other sick animals.	

**RISK PERCEPTIONS**	
Have you heard that bush animals may give people disease?	No<br>Yes
Do you believe animals give you diseases?	Strongly disagree<br>Disagree<br>Neither agree nor disagree/do not know<br>Agree<br>Strongly agree
Are you afraid of this?	Very unafraid<br>Unafraid<br>Neither agree nor disagree/do not know<br>Afraid<br>Very afraid
How do you think that people get sick from animals?	Doesn't believe<br>Direct contact during hunting<br>Direct contact during processing or cooking<br>Consumption<br>Proximity<br>Breathing the same air

	Indirect contact<br>Other

Household Economics

Enumerate the livestock this household owns.

Species	Total
Cattle	
Pigs	
Dogs	
Goats	
Chickens	
Sheep	
Ducks	
Other	

Do you have electricity?	No<br>Yes
If yes, what is the source of your electricity? *skiplogic*	Connection (to grid)<br>Solar panels<br>Generator

Enumerate the amenities this household owns.

Item	Total
Radio	
Television (screen)	
Computer	
Refrigerator	
Table	
Chair	
Bed	
Sofa	
Cupboard	
Video player	
Air conditioner	
Electric iron	
Generator	
Fan	
Satellite dishes	
Big solar panels	
Large solar panels	
Car	
Watch	
Mobile phone	

https://doi.org/10.3390/ani16111666

Bicycle	
Motorcycle/scooter	
Boat with a motor	
Canoe	
Keke Napep/ tricycle	

What is your highest level of education?	None<br>Primary school<br>Secondary school<br>Tertiary school
If tertiary, what kind of tertiary school?<br>*Select multiple.*<br>*Skip logic: If Tertiary School= yes*	Diploma<br>College of Education<br>Polytechnic<br>University

On average, how much money do you make from bushmeat in a month? *This excludes expenditures*	(# naria)
Is this your primary source of income?	No<br>Yes
Do you have written records of sales at this location (at least the prior week)?	No<br>Yes
If yes, may we see them and take a picture?<br>*Note the file names of the pictures*	No<br>Yes

Add any other notes here	

https://doi.org/10.3390/ani16111666

Supplementary Materials S2: Monthly Survey

Observed Meat— First, ask the interviewee about each item of bushmeat that is present and document their responses to questions 1-8 in the table below.

If they respond "other" to a question, write the question number and explanation in the space for "Notes" below that row. Write observed vs recall in row notes.

Each row represents one item of bushmeat.

1. What is the species? *Select one Reference the species photos and enter the number of the corresponding species or Unknown (UN); Not applicable (NA).*
2. What body part? *Select multiple* Whole or >50% carcass (W50); Hand (HA); Lap (LA); Trunk (TK); Head + Neck (HN); Internal organs (IO); (UN); (NA)
3. How did you acquire it? *Select one* Hunted – self (HS); Hunter – other (HO); Household (HH); Middleman (MM); Vendor - Mobile (VO); Vendor - Market (VM); Vendor - Roadside (VR); Restaurant (RE); Other (OT); (UN); (NA)

3.1 If you captured it, did you set out to catch it or did you encounter it opportunistically. *Select one* Targeted hunting (TH), Opportunistic – Alive (OA); Opportunistic – Dead (OD); (OT); (UN); (NA)

4. 3.2 If you captured it, how did you kill that animal: *Select multiple* Gun (GU); Trap (TP); Dogs (DO); Snares (SN); Spears (SP); Machete (MA); Poison (PO); (OT); (UN); (NA)
5. What area did it come from? *Select one* Oban Division (OB); Cameroon (CM); (OT); (UN); (NA)
6. How was it processed when you first see it? *Select multiple* Alive (AL); Whole - Dead (WO); Gutted (organs removed) (GT); Cleaned (CN); Scalded (SD); Butchered (into parts) (BA); Butchered (into pieces) (BI); Roasted (RO); (UN); (NA)
7. How was it preserved when you first see it? *Select one* Alive (AL); Raw (RA); Parboiled (steamed) (PB); Smoked (pink inside) (SM); Dried (no pink inside) (DR); Cooked (boiled, bbq) (CK); Frozen (FR); Salted (SA); (UN); (NA)
8. For the meat that is not consumed, how have you processed it? *Select multiple* Gutted (organs removed) (GU); Cleaned (CN); Scalded (SD); Butchered (into parts) (BA); Butchered (into pieces) (BI); Roasted (RO); Discarded parts (DI); No action (NA); (UN)
9. For the meat that is not consumed, how have you preserved it? *Select multiple* Parboiled (steamed) (PB); Smoked (pink inside) (SM); Dried (no pink inside) (DR); Cooked (boiled, bbq) (CK); Froze (FR); Salted (SA); No action (NA); (UN)

***Recalled Meat*—Now, tell me about any other bush animals you interacted with in the prior week.**

This includes any time you were touching, trading, cooking, consuming, interacting with bushmeat or live wild animals including pets?

Repeat questions 1-8 then move on to questions 9-11

9. Where did it go? *Select one* Consumer - self (CS); Consumer – others (CO); Hunter (HU); Middleman (MM); Vendor - Mobile (VO); Vendor - Market (VM); Vendor - Roadside (VR); Restaurants (RE); (OT); (UN); (NA)

10. Was this meat: *Select multiple* Sold (SO); Exchanged (EX); Gifted (GI); Consumed here (CH); Used for zootherapy (ZO); Discarded (DI); (OT); (UN); (NA)

https://doi.org/10.3390/ani16111666

Row Notes: If used for Zootherapy, what was the purpose and how was it administered. If Discarded is selected, document the reason.

11. For the meat you have not consumed, how many <u>hours</u> was it in your possession (from first touch point to last)?

***ALL above meat* — Now we are going to talk about all of the animals you interacted with in the past week, <u>and</u> any of the meat here with us now.**

12. Were any of these animals sick? If yes, which and how did you know it was sick? *Select multiple* Lesions (LE); Growths (GR); Emaciated (EM); Wound/Injury (WI); Worms (WO); Ecotoparasites: Tumbu fly, ticks (EC); Behavioral of animal (BE); Color of the meat (CL); Endoparasite (EN); (OT); (UN); (NA)

Row Notes: use this space to provide any additional details (very important).

13. Did you ever get hurt interacting with any of them? If yes, how? *Select multiple* Bitten (BT); Scratched (SC); Punctured (PU); Cut (CT); (OT); (UN); (NA)

13.1 Rank the severity of the injury. *Add the number after the letter from 13* 1 minor (self-treated); 2 moderate (visited chemist); 3 severe (hospital care)

14. Did children interact with any of these animals? *Select multiple* Hunt (HT); Transport (TR); Play (PL); Processed (PR); Preserved (PE); Sell (SE) Touched (TO); (OT); (UN); (NA)

15. If you have sold this meat, what was the price (naira)? (UN); (NA)

IMPORTANT: ***Mark any parts that were sampled by writing the sample number in SMPL***

Supplementary Materials S3

Table S1. Trade-role differences in hygiene and precautionary behaviors during game meat handling, based on Kruskal–Wallis tests.

Precaution	Chi-Squared	df	P-Value	Significance
Wash hands before handling meat	4.055701	2	0.1316	
Wash hands before handling meat with soap	5.336264	2	0.0694	
Wash hands after handling meat	9.729462	2	0.0077	**
Wash hands after handling meat with soap	23.006284	2	0.0000	***
Use body protection while handling meat	6.777243	2	0.0338	*
Change clothes after handling meat	18.046222	2	0.0001	***
Wash containers used for meat	17.123322	2	0.0002	***
Wash containers used for meat with soap	44.849344	2	0.0000	***
Wash surfaces used for meat	14.577321	2	0.0007	***
Wash surfaces used for meat with soap	32.976538	2	0.0000	***
Wash tools used for meat	14.447598	2	0.0007	***
Wash tools used for meat with soap	51.145484	2	0.0000	***
Take measures to prevent meat spoiling	7.696637	2	0.0213	*
Cook meat well	6.558664	2	0.0377	*
Avoid using same surfaces for meat and non-meat items	2.727145	2	0.2557	
Avoid using same tools for meat and non-meat items	1.374731	2	0.5029	

https://doi.org/10.3390/ani16111666

Precaution	Chi-Squared	df	P-Value	Significance
Avoid using same containers for meat and non-meat items	14.134720	2	0.0009	***
Avoid consuming pink meat	2.521911	2	0.2834	
Avoid consuming raw meat	1.434759	2	0.4880	

Table S2. Dunn's post-hoc pairwise comparisons of trade-role differences in hygiene and precautionary behaviors during game meat handling.

Precaution	Comparison	Z-Score	Adjusted P-Value	Significance
Wash hands after handling meat	Restaurants - Vendors	2.635398	0.0126	*
Wash hands after handling meat with soap	Hunters - Restaurants	-4.597076	0.0000	***
Wash hands after handling meat with soap	Restaurants - Vendors	2.175917	0.0443	*
Use body protection while handling meat	Hunters - Restaurants	-2.483916	0.0195	*
Change clothes after handling meat	Hunters - Restaurants	-3.829307	0.0002	***
Change clothes after handling meat	Restaurants - Vendors	2.500160	0.0186	*
Wash containers used for meat	Hunters - Restaurants	-4.076590	0.0001	***
Wash containers used for meat with soap	Hunters - Restaurants	-6.578376	0.0000	***
Wash containers used for meat with soap	Hunters - Vendors	-2.303189	0.0319	*
Wash containers used for meat with soap	Restaurants - Vendors	2.421482	0.0232	*
Wash surfaces used for meat	Hunters - Restaurants	-3.817633	0.0002	***
Wash surfaces used for meat with soap	Hunters - Restaurants	-5.521932	0.0000	***

**Precaution**	**Comparison**	**Z-Score**	**Adjusted P-Value**	**Significance**
Wash surfaces used for meat with soap	Restaurants - Vendors	2.547122	0.0163	*
Wash tools used for meat	Hunters - Restaurants	-3.059911	0.0033	**
Wash tools used for meat	Restaurants - Vendors	2.770160	0.0084	**
Wash tools used for meat with soap	Hunters - Restaurants	-7.034241	0.0000	***
Wash tools used for meat with soap	Hunters - Vendors	-2.506843	0.0183	*
Wash tools used for meat with soap	Restaurants - Vendors	2.538786	0.0167	*
Take measures to prevent meat spoiling	Hunters - Restaurants	-2.764167	0.0086	**
Cook meat well	Hunters - Restaurants	-2.211024	0.0406	*
Avoid using same containers for meat and non-meat items	Hunters - Restaurants	-3.609632	0.0005	***
